# MicroRNA signature refine response prediction in CML

**DOI:** 10.1038/s41598-019-46132-9

**Published:** 2019-07-04

**Authors:** Raquel Alves, Ana Cristina Gonçalves, Joana Jorge, Gilberto Marques, Dino Luís, André B. Ribeiro, Paulo Freitas-Tavares, Bárbara Oliveiros, António M. Almeida, Ana Bela Sarmento-Ribeiro

**Affiliations:** 1 Laboratory of Oncobiology and Hematology and University Clinic of Hematology/Faculty of Medicine, University of Coimbra (FMUC), Coimbra, Portugal; 2 Coimbra Institute for Clinical and Biomedical Research (iCBR) - Group of Environment Genetics and Oncobiology (CIMAGO), FMUC, Coimbra, Portugal; 3 Center for Neuroscience and Cell Biology (CNC.IBILI), University of Coimbra, Coimbra, Portugal; 4 Clinical Pathology Service, Centro Hospitalar Universitário de Coimbra (CHUC), Coimbra, Portugal; 5 Clinical Hematology Department, CHUC, Coimbra, Portugal; 6 Laboratory of Biostatistics and Medical Informatics, Faculty of Medicine, University of Coimbra, Coimbra, Portugal; 7 Hospital da Luz, Lisbon, Portugal; 8 CIIS (Centro de Investigação Interdisciplinar em Saúde) Universidade Católica Portuguesa de Lisboa, Lisbon, Portugal

**Keywords:** Chronic myeloid leukaemia, Tumour biomarkers

## Abstract

microRNAs (miRs) dysregulation have emerged as a crucial step in tumorigenesis, being related with cancer development, progression and response to treatment. In chronic myeloid leukaemia (CML), the resistance to tyrosine kinase inhibitors (TKI) is responsible for treatment failure and could be linked to changes in miRs expression. This work aimed to correlate the expression levels of 3 miRs, miR-21, miR-26b and miR-451, with response to TKI treatment in CML patients. miR-451 levels at diagnosis were significantly higher in patients with optimal response after 6 and 12 months of therapy. Conversely, patients without optimal response had highest levels of miR-21. miR-21 and miR-451 appear to be good biomarkers of response, able to predict optimal TKI responders (p < 0.05). Using the combined profile of both miRs, we create a predictive model of optimal response after one year of treatment. This study highlights the role of miR-21 and miR-451 expression levels at diagnosis in predicting which patients achieve the optimal response.

## Introduction

MicroRNAs (miRs) are a group of small single-strained non-coding RNAs (≈22 nucleotides of length) that act as epigenetic regulators of specific targets by modulating gene expression via translational repression or mRNA cleavage^1^. Although protein inhibition is the expected result of miR function, this is not always an inhibitory process. miRs can indirectly activate the expression of some mRNAs by degradation of their natural inhibitors^1,2^. These small RNAs regulate around 80% of the transcriptome and play a key role in multiple cellular processes, like proliferation, development, differentiation and apoptosis^2^.

The dysregulation of miRs expression patterns can have numerous implications including the promotion of tumorigenesis^3^. miRs expression pattern differs according to tissues, cell types and developmental stages, and could act as oncomiRs, promoting processes that favour cancer cells, or as tumour suppressor miRs, regulating the expression of oncogenes^4,5^. Aberrant expression of miRs has been reported in solid tumours and haematological neoplasia, as chronic myeloid leukaemia (CML)^6,7^. The presence of *BCR-ABL1* is the distinctive molecular characteristic of CML and the target for treatment with tyrosine kinase inhibitors (TKI)^8^. Resistance to imatinib and other TKIs has been recognised as the major challenge for CML treatment and monitoring, since some of the imatinib-resistant patients had no mutations on *BCR-ABL1* oncogene^9^. Since miRs are potent regulators, they may be implicated in the acquisition of drug resistance because they could regulate not only *BCR-ABL1* but also modulate the expression of genes involved in drug transporter or activation of essential signalling pathways^6,10^. In CML the most deregulated miRs studied include miR-10a, miR-130b, miR-150 and miR-203, but several others miRs emerge in the complex network of miR regulation^11,12^.

Overexpression of the oncomiR miR-21 has been associated with the development of neoplasias where it targets many tumour suppressor genes related to proliferation, apoptosis and survival^13^. One example of miR-21 targets is PTEN, a negative regulator of the PI3K/AKT pathway, which is often described as deregulated in cancer. In addition, levels of miR-21 were correlated with acquisition of resistance to multiple drugs, as gemcitabine and TKIs^13,14^. PTEN is also a target of miR-26b, but the role of this miR in cancer is still controversial, being sometimes described as oncomiR and others as a tumour suppressor miR^15,16^. Particularly relevant in CML are the miRs that target *ABL* and consequently *BCR-ABL1*, being some of the most deregulated ones. miR-451, miR-203 and miR-320a are described as downregulated in CML, since they directly target *ABL* and *BCR-ABL1* acting as tumour suppressors, by reducing BCR-ABL oncoprotein expression^12,17^.

Furthermore, miRs expression is a dynamic process which reflects changes at cellular levels not only at the time of neoplasia development but also in progression or response to treatment. These characteristics made the expression levels of miR a potential biomarker for diagnosis, prognosis and treatment response^18,19^.

In this work, we analysed the expression levels of miR-21, miR-26b and miR-451 in CML patients and *in vitro* models, and correlated them with TKI response levels. The potential of each miR as a predictive biomarker was explored, with a particular emphasis for the combinatorial miR expression profile.

## Results

### Differential miRs expression levels at Imatinib-resistant models

**Figure 1:**
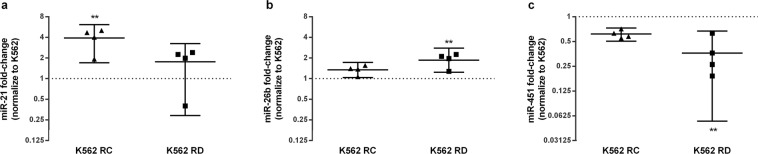
miRs expression profile of imatinib-resistant CML cell lines. Sensitive cell line, K562 cells, was used as reference to determine the fold-change of miR-21 (**a**), miR-26b (**b**) and miR-451 (**c**) of resistant cell lines, K562-RC and K562-RD cells. miR-21 was higher in K562-RC, while miR-26b was higher in K562-RD. Moreover, miR-451 was significantly down-regulated in K562-RD. The results are presented in mean with 95% CI of four independent samples and the expression of K562 cell line is represented by dot line. **p < 0.001 compared with K562 cells.

To check if the expression levels of miR-21, miR-26b and miR-451 may be related with Imatinib resistance, we evaluated the levels of each miR in three CML cell lines (one sensitive and two resistant to TKI, the K562-RC and the K562-RD cells respectively). The Imatinib-resistant cells presented different expression levels of miRs compared to the sensitive cell line (Fig. 1). The oncomiR miR-21 was up-regulated in both resistant models, with 4.3-fold higher expression in K562-RC cells (p = 0.0038; Fig. 1a). Similarly, miR-26b was increased in resistant cells, with 2.0-fold higher expression in K562-RD cells (p = 0.0023; Fig. 1b). By opposition, the tumour suppressor miR-451 was down-regulated in K562-RC and K562-RD cells, with 3.8-fold less expression in the discontinuation model (K562-RD) (p = 0.0048; Fig. 1c).

### CML patients miRs expression levels at diagnosis

**Figure 2:**
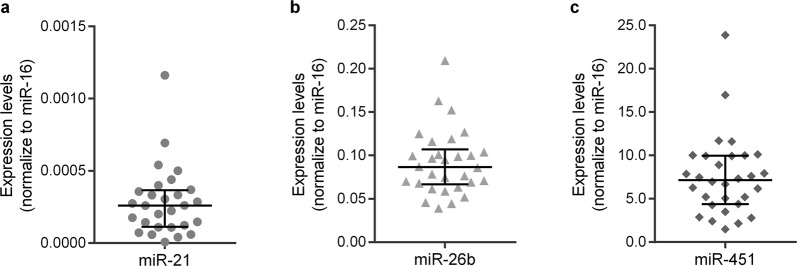
miRs expression levels in CML patients at diagnosis. The expression levels of miR-21 (**a**), miR-26b (**b**) and miR-451 (**c**) were normalised to miR-16 in CML patients at diagnosis time. At diagnosis, miR-451 was the miR with the highest expression and miR-21 with the lowest. The results are represented as median with interquartile range.

We subsequently assessed the expression of the same miRs in CML patients, samples at diagnosis (Fig. 2). Clinical and biological characteristics of our cohort of patients were summarised in Table 1. miR-26b and miR-451 were expressed in all patients, the tumour suppressor miR-451 exhibiting the highest expression at diagnosis [7.147 (95% confidence interval (CI): 5.201–8.912; Fig. 2c)], and the miR-26b the second one with a median of 0.087 (95% CI: 0.070–0.099; Fig. 2b). miR-21 was expressed in 93.3% of patients (28 out of 30 patients) with a median of 0.003 (95% CI: 0.00014–0.00033; Fig. 2a). The expression of this miR was inversely correlated with the expression of miR-451 (R = −0.655; p = 0.0002). miR-26b expression was positively correlated with miR-21 expression (R = 0.611; p = 0.001) and inversely associated with miR-451 (R = −0.462; p = 0.010).

**Table 1:** Biodemographic and clinical characteristics of CML patients.

Characteristics	Diagnosis group (n = 30)		Follow-up group (n = 27)	
**Demographic features**				
Gender (%)				
Male	16	(53.3)	14	(51.9)
Female	14	(46.7)	13	(48.1)
**Clinical features**				
Age at diagnosis (years)				
Median	54	42		
Range	18–78	24–78		
Disease Phase				
Chronic Phase (%)	25	(83.3)		
Accelerate Phase or Blast Crisis (%)	5	(16.7)		
Treatment			*at analysis time point*	
TKI (%)	27	(90.0)	22	(81.5)
Imatinib (%)	26	(96.3)	14	(63.6)
2^nd^ TKI (%)	1	(3.7)	8	(36.4)
TKI plus INF-α (%)	3	(10.0)	5	(18.5)
Imatinib (%)	2	(66.7)	4	(80.0)
2^nd^ TKI (%)	1	(33.3)	1	(20.0)
Response	*at 6 Months*		*at analysis time point*	
Optimal Response (%)	17	(56.7)	24	(88.9)
Without Optimal Response (%)	9	(30.0)	3	(11.1)
No determined (%)	4	(13.3)		
	*at 12 Months*			
Optimal Response (%)	15	(50.0)		
Without Optimal Response (%)	14	(46.7)		
No determined (%)	1	(3.3)		

TKI: Tyrosine kinase inhibitors; INF-α: interferon-alpha.

The observed miR expression levels may be related with the disease phase at diagnosis, since miR-451 shown a tendency to be downregulated at accelerated phase or blast crisis [median of 4.28, Interquartile Range (IR) 1.82–8.92, n = 5)] in comparison with patients in chronic phase (median of 7.31, IR 5.13–10.02, n = 25). However, the low number of patients in advanced phases did not allow statistically significant differences to be detected. For that it would be necessary at least 68 patients in each group (G power analysis with 80% of power).

### TKI Response and miRs expression levels

**Figure 3:**
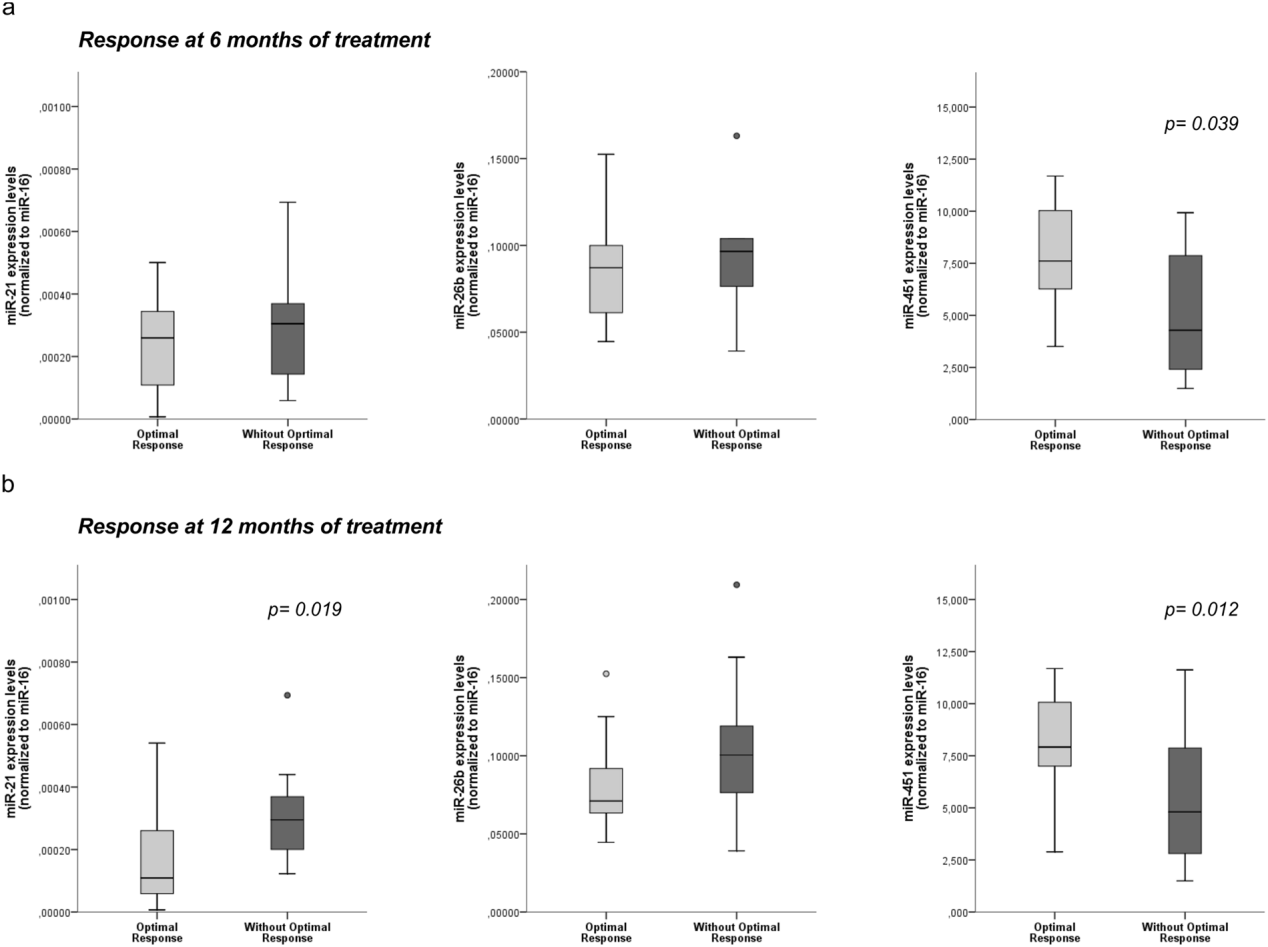
Correlation between TKI response and miRs expression levels at diagnosis. Patients were grouped according to TKI response in optimal response or without optimal response, reached after 6 and 12 months of treatment. The expression levels of miR-21, miR-26b and miR-451 at diagnosis were analysed according to the TKI response after 6 (**a**) and 12 (**b**) months of treatment. miR-451 was down-regulated in patients without optimal response after 6 and 12 months of TKI therapy, while miR-21 was up-regulated in patients without optimal response after 12 months of treatment. Statistical differences between groups are reported by the p-value.

To confirm if the miRs expression at diagnosis could be related with the degree of response achieved after TKI treatment, we evaluated the expression profile according to response levels after 6 and 12 months of treatment^20^. Optimal response was considered when *BCR-ABL1* expression levels were lower than 1% at 6 months and ≤0.1% at 12 months of treatment^20^. Patients with higher levels of miR-451 at diagnosis had a higher rate of optimal response after 6 months (p = 0.039; Fig. 3a). Our results reveal that the lower expression of miR-21 (p = 0.019) and higher levels of miR-451 (p = 0.012) were associated with patients with optimal response at 12 months of treatment (Fig. 3b). At both time points, the levels of miR-26b were higher in patients without optimal response, but the differences were not statistically significant.

**Figure 4:**
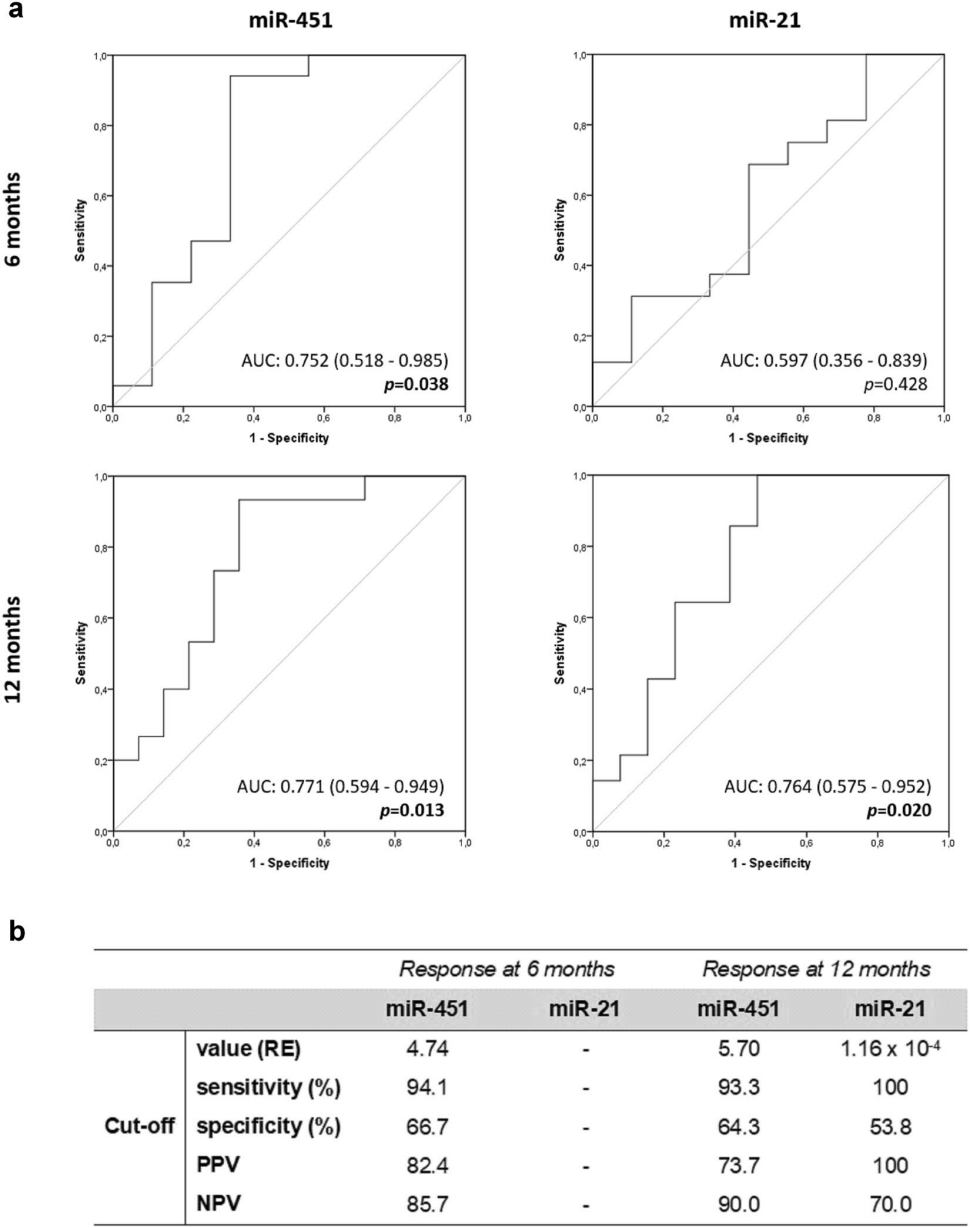
Receiver Operating Characteristic (ROC) curve analysis of miR-451 and miR-21. ROC curves (**a**) and the identification of optimal cut-off values to discriminate optimal response (**b**) point-out the ability of miR-451 and miR-21 expression at diagnosis to predict response to TKI treatment at 6 months and 12 months. For each curve was represent the Area Under the Curve (AUC) and the respective p-value. RE–Relative Expression; PPV- Positive Predictive Value; NPV- Negative Predictive Value.

To explore the predictive power of miRs expression to predict response to TKI treatment, we performed ROC curve analysis (Fig. 4). miR-451 expression discriminated patients with optimal response from those without response after 6 months of treatment (p = 0.038; Fig. 4a). In addition, both miR-451 and miR-21 revealed significant potential as response biomarkers at 12 months of TKI (Fig. 4a). The expression of miR-451 was the most accurate parameter to predict response with an area under the curve (AUC) of 0.771 (95%CI: 0.594–0.949; p = 0.013). At diagnosis, miR-451 expression levels higher than 4.74 and 5.70 were the optimal cut-off values to discriminate optimal response after 6 and 12 months of treatment, respectively (Fig. 4b). These cut-off values present higher sensitivity, 94.1% at 6 months and 93.3% at 12 months, with a satisfactory specificity of 66.7% and 64.3% for both time points. Conversely, levels of oncomiR miR-21 below 1.16 × 10^−4^ at diagnosis was identified as the best cut-off value for optimal response at 12 months of treatment. This biomarker revealed the highest sensitivity (100%) and also a strong positive predictive value (PPV:100%) and a good negative predictive value (NPV: 70%) (Fig. 4b).

### miRs profile and response after a year of treatment

Since we identified both miR-21 and miR-451 as good biomarkers of response after a year of treatment with TKI, we made a profile analysis and risk association. Using the optimal cut-off values detected for each miR, we classified patients in four different profiles according to miR-21 and miR-451 expression levels: Profile 1: miR-451 > 5.69 and miR-21 < 1.16 × 10^−4^; Profile 2: miR-451 < 5.69 and miR-21 > 1.16 × 10^−4^; Profile 3: miR-451 > 5.69 and miR-21 > 1.16 × 10^−4^; Profile 4: miR-451 < 5.69 and miR-21 < 1.16 × 10^−4^. After, we correlated these miRs profiles with the probability to achieve an optimal response after 12 months of treatment. All patients included in the first profile presented an optimal response (8 optimal responses out of 8), while most of those in profile 2 do not reach optimal response (1 optimal response out of 10). Risk analysis showed that profile 2 patients have 25 times lower probability to achieve optimal response after one year of treatment (OR: 0.040; 95%CI: 0.0039–0.398; p = 0.001). On the other hand, patients with profile 1 presented 42 times higher chances to accomplish an optimal response at the same time point (OR: 42.38; 95%CI: 2.129–843.9; p = 0.0007). Profile 3 was not statistical associated with response (4 optimal responses out of 9), and no patient presented profile 4.

In this context, miR-21 and miR-451 levels at diagnosis were used to create a predictive model for an optimal response after one year of treatment. From the different regression models tested, the main effect (ME) model that takes in consideration both miRs for optimal response, was the most predictive one accounting for a total of 66% of the variance (Table 2). Applying this model, we will be able to assess the individual probability of each patient to achieve an optimal response (P) Eq. (1).

1 $$P(OptResp)=\frac{1}{1+{e}^{-(19,6-19,6\times miR21-2,2\times miR451)}},$$

In Eq. (1), miR21 and miR451 represent the miR expression in a dichotomized form (miR-21 ≤ 1.16 × 10^−4^ = 0 and miR-21 > 1.16 × 10^−4^ = 1; miR-451 > 5.7 = 0 and miR-451 ≤ 5.7 = 1).

**Table 2:** Statistical measures for predicting models for optimal response.

Model	Varibles dichotomize			
	miR-21	miR-451	Interaction	Main Effect
Raw Aggrement (%)	79.31	79.31	79.31	79.31
kappa (Cohen)	0.59	0.58	0.58	0.58
Sensitivity (%)	100.00	64.29	64.29	64.29
Specificity (%)	60.00	93.33	93.33	93.33
PPV (%)	70.00	90.00	90.00	90.00
NPV (%)	100.00	73.68	73.68	73.68
acuracy (Youden)	60.00	57.62	57.62	57.62
R^2^ (Nagelkerke)	0.56	0.45	0.45	0.66
AIC	28.44	32.40	32.40	26.36

PPV: Positive predictive value; NPV: Negative predictive value; AIC: Akaike information criterion.

### miRs expression at follow-up

**Figure 5:**
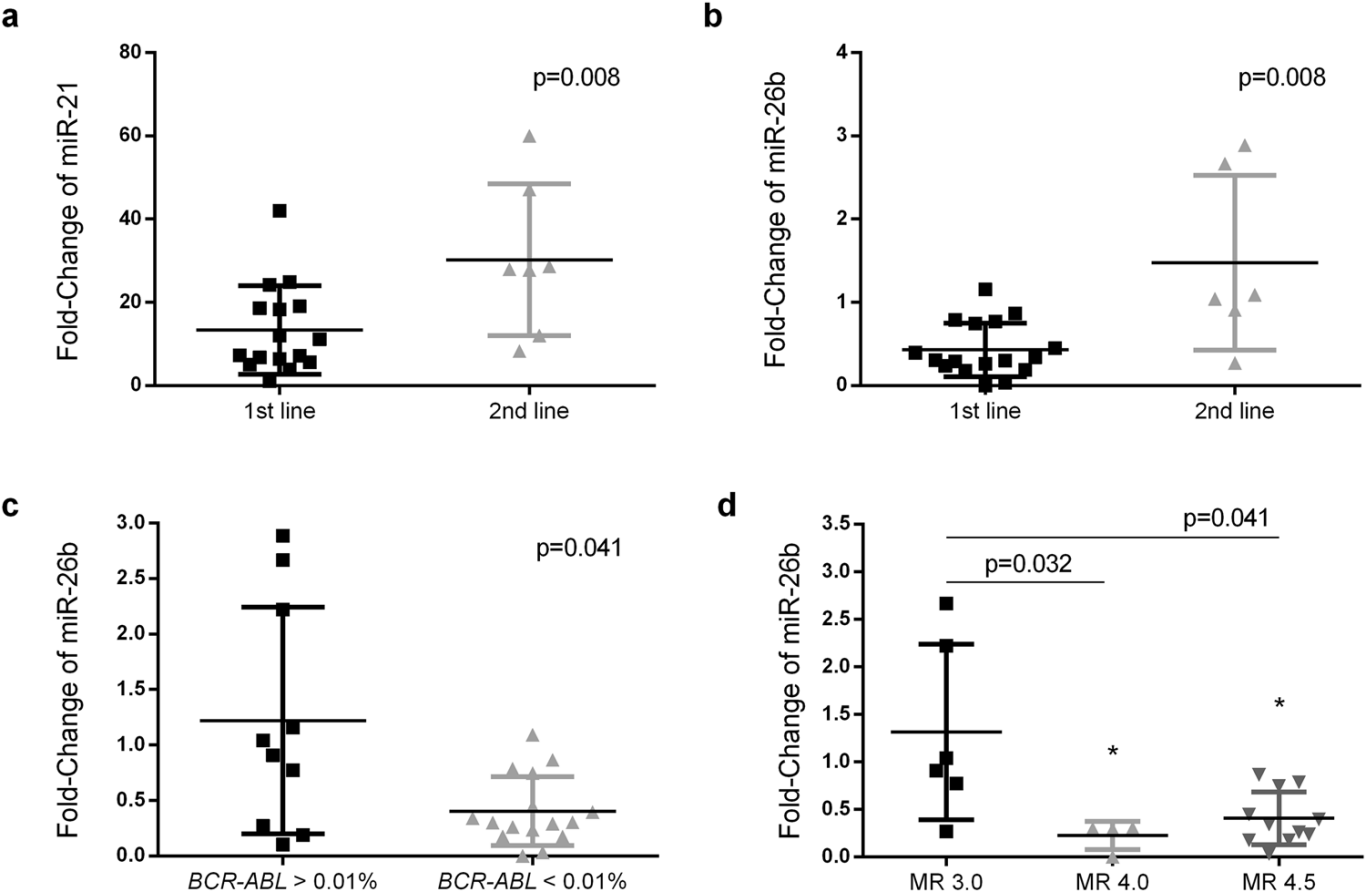
Analysis of miR-21 and miR-26b fold-change in patients under TKI treatment. miR-21 (**a**) and miR-26b (**b**) fold-change were analysed according to the number of TKI that patients were exposed. The miR-26 expression levels were also studied in patients stratified according to *BCR-ABL1* values (**c**) and to molecular response rates (**d**) Higher levels of miR-21 and miR-26b were detected in patients that fail 1^st^ line of treatment, whereas miR-26b were down-regulated in patients with lower *BCR-ABL1* levels and also in patients with deeper responses to treatment (MR 4.0 and MR 4.5). Statistical differences between groups are reported by the p-value.

Due to lack of samples, it was not possible to determine the miRs expression at diagnosis in the follow-up group. To determine the fold-change of this group, we used the median of each miR observed in the diagnosis group as a reference, since the values did not present a normal distribution. mir-21 was up-regulated 15.0 times during the treatment, while miR-26b and miR-451 were down-regulated 2.4 and 9.2 times, respectively. Correlating the expression levels with clinical and laboratory data, we observed higher levels of miR-21 (p = 0.008; Fig. 5a) and miR-26b (p = 0.008. Figure 5b) in patients that fail 1^st^ line of treatment and switch for other TKI. Additionally, miR-26b levels were significantly downregulated in patients with lower *BCR-ABL1* quantification (p = 0.041; Fig. 5c). Furthermore, we observed that patients with a deeper molecular response (MR) presented lower levels of miR-26b than those with MR of 3.0 (MR 4.0 p = 0.032. and MR 4.5 p = 0.041) (Fig. 5d).

## Discussion

Most deregulated miRs act on specific signalling pathways that seem to be essential to promote and maintain cancer cell survival and growth^4^. This critical regulatory role, led to associations between miRs expression and response to anticancer drugs proposed by several authors^10^. Our results from *in vitro* models highlighted the relation of miR-21 and miR-26b overexpression and miR-451 downregulation with imatinib-resistant phenotype. As expected, an unbalance between oncomiRs and tumour suppressor miRs were observed in the resistant models. In line with *in vitro* models results, we found a correlation between miR-21 and miR-451 expression at diagnosis with the response to TKI treatment. In our cohort, we observed that the optimal response to TKI after 6 months was related to high levels of miR-451. After 12 months of treatment, patients with an optimal response presented not only high levels of miR-451 but also low levels of miR-21, compared to those without optimal response.

miR-451 acts as a tumour suppressor and has been reported to be downregulated in some neoplasias, such as gastric cancer, glioblastoma and leukaemia^21–23^. Particularly in CML, the expression of miR-451 was associated with a favourable prognosis, since this miR targets *ABL* and *BCR-ABL1* directly^12^. According to Lopotová *et al*.^24^, the miR-451 expression is downregulated in CML at diagnosis compared with healthy controls, and this could be due to BCR-ABL activity^25^. The same authors suggested a reciprocal regulatory loop between this miR and *BCR-ABL1* levels that could be interrupted by imatinib treatment^24^. This interaction could have a critical impact on response level, and levels of miR-451 constitute an excellent marker to predict response to TKI. In our results, miR-451 levels were able to predict patients that will achieve response at an early point (6 months) and after one year of treatment, with very good sensitivity and specificity. In the same line, Su *et al*. (2015) suggested miR-451 as a potential prognostic biomarker in gastric cancer^23^. The introduction of new biomarkers, as could be the case of miR-451, will improve patient monitoring and could have an impact on therapeutic selection.

With opposite functions, miR-21 is one of the most overexpressed miRs in solid and haematological tumours^13^. We observed that low levels of this miR at CML diagnosis were related with the achievement of optimal response to TKI therapy. miR-21 present multiple targets as PTEN, PDCD4 and TIMPs that contribute to the promotion of proliferation, survival and invasion pathways, however according to cell type, the principal mechanism of action may be different^13,26^. In some cells, it could be related to the up-regulation of BCL-2 to prevent apoptosis, while in others the most significant effect may be the inhibition of PTEN and consequently the activation of PI3K/AKT pathway^13,27^. In CML, BCR-ABL, with its constitutive tyrosine kinase activity, stimulates multiple signalling pathways such as PI3K/AKT and MAPK/ERK. The downregulation of PTEN and inhibition of PDCD4 has been described as the most crucial action of miR-21 in CML cells^26,28^. The correlation between miR-21 expression and resistance to anticancer agents, describe by us and other groups, highlight the possible clinical application of this miR as a target for therapy^29^. Several groups showed an increase in cell death and a synergistic effect with other drugs when miR-21 was inhibited by antagomiRs or some molecules that modulate miR-21, as curcumin^30–32^. In this work, we did not test miR-21 as a therapeutic target, but we assess the power as a tool for predicting a good response to TKI treatment. Levels of miR-21 measured at diagnosis work as a biomarker of response after one year of TKI therapy. In childhood B cell acute lymphoblastic leukaemia, the same miR is an independent prognostic marker, were high levels were associated with poor response and shorter overall survival^33^.

Using a single measure to predict prognosis, progression, response and many other aspects seem very simple and sometimes difficult to explain the cell complexity. Based on this, we look for the combinatorial miR expression profile since two miRs showed biomarkers characteristics. Based on cut-off values identified for miR-21 and miR-451, we created 4 different profiles to assess the risk/ probability to achieve optimal response after 12 months of TKI treatment. Patients with a high level of miR-451 and low levels of the oncomiR miR-21 at diagnosis (profile1) presented 42 times more probability of achieving an optimal response. According to miRs functions, profile 1 appears as the best combination possible supporting a more controlled environment against cancer cells growth. In opposition, profile 2, which comprehend higher oncomiRs expression, lead to a very low probability of these patients reaching optimal response.

By identifying patients at high risk of relapse or progression under treatment, we can prevent several complications and disease progression. Improving the disease monitoring or introducing changes in therapeutic options for these subgroups of patients could be the best approach to achieve better results. These predictive models might play an important role in identifying subgroups of patients based on clinical and biological features. Sokal, EURO, EUTOS and ELTS are scores used in CML for discriminating overall survival (Sokal and EURO), predicting complete cytogenetic remission (CCgR) 18 months after the start of therapy, and predict the probability of CML related death, respectively^34–37^. These scores are based on age, spleen size and haematological parameters at diagnosis, such as basophils, blast cells and platelets. However, none of them were designed to predict optimal response to TKI treatment. Using our miRs results, we create a predictive model (Eq. 1) to assess the probability of each patient achieving and optimal response to TKI treatment after one year of therapy. The identification of new biomarkers of response/resistance to TKI is in constant progress, and several molecules present good discriminating power, namely specific gene signatures. Nevertheless, independent validation of all new biomarkers and scores would be very helpful to implement in the clinic the most accurate one to predict the optimal response.

In order to increase the number of patients studied and due to the impossibility of obtaining samples at diagnosis from all patients, we use the medians of miRs expression at diagnosis to estimate the fold-change in miR expression induced by TKI treatment. Although not statistically significant, the expression levels of miR-26b at diagnosis was higher in patients that did not reach optimal response with TKI. However, this miR presented some relevance at follow-up measures in a group of treated patients. The majority of studies describe miR-26b as a tumour suppressor miR that can inhibit cell proliferation in breast cancer, hepatocellular carcinoma and cervical cancers^16,38,39^. However, some contradictory results have suggested that this miR could act as oncomiR. Palumbo *et al*.^15^ revealed that the regulation of PTEN in pituitary tumour cells is mediated by miR-26b action^15^. miR-26b by targeting PTEN, as also describe to miR-21, leave the PI3K/AKT signalling pathway without regulation, promoting tumorigenesis. In agreement with these authors, our results suggest that lower levels of miR-26b were associated with a good response to the first line of treatment and with more profound responses. These results suggest that miR-26b might be an indicator of good responses levels.

The link between miR-21, miR-26b and miR-451 and TKI response levels need to be validated in a bigger cohort of patients before moving into clinical practice. On the other hand, understanding the crucial role of these miRs in disease progression and treatment response might be very useful for exploring these miRs as a new therapeutic target in CML. In conclusion, expression levels of miR-21 and miR-451 at diagnosis play a crucial role in CML response to TKI treatment and may constitute a new potential biomarkers of response and for the guidance of therapeutic options.

## Material and Methods

### Cell lines

K562 cells sensitive to Imatinib and two Imatinib-resistant models (K562-RC and K562-RD cells) were used as *in vitro* models of CML. The sensitive cell line was obtained from ATCC, and the Imatinib-resistant cell lines were developed in our laboratory based on two strategies: a continuous exposure (K562-RC) and a discontinuous exposure to Imatinib (K562-RD), as described in Alves *et al*.^40^.

### Study population

Fifty-seven CML patients were enrolled in the present study and recruited at Clinical Hematology Department of Centro Hospitalar Universitário de Coimbra (CHUC), Portugal. Patients were grouped according to different time points: at diagnosis (diagnosis group, n = 30) and follow-up (follow-up group, n = 27). Clinical and biological characteristics were summarised in Table 1. Treatment response criteria were defined according to the European Leukemia Net (ELN) guidelines^20^, where optimal response corresponds to *BCR-ABL* levels lower than 1% at 6 months and ≤0.1% at 12 months. Patients that presented levels higher than the established cut-off were incorporated in “without optimal response” group. The study was conducted according to the Helsinki Declaration, and all participants provided informed consent for participation before enrolment. The Ethics Committee of the Faculty of Medicine (University of Coimbra, Portugal) approved all research procedures (ref. CE-014/2014).

### RNA extraction, cDNA synthesis, and real-time PCR

Total RNA was extracted from CML patients’ samples and cell lines using the mirVana miRNA Isolation Kit (Ambion), according to the manufacturer’s protocol. RNA quantity and quality were measured using a NanoDrop ND-1000 spectrophotometer. TaqMan® Advanced miRNA cDNA Synthesis Kit (Applied Biosystems) was used to convert 10 ng of total RNA, according to the manufacturer’s protocol. Briefly, this kit included several steps: the Poly(A)-tailing reaction, the ligation reaction, reverse transcriptase reaction followed by a miR-Amp reaction. cDNA was diluted to 1:10 with DNase/RNase free water before use. The levels of miR-21, miR-451, miR-26b and miR-16 were determined in each sample. The quantitative real-time polymerase chain reaction (qPCR) was performed using TaqMan Advanced miRNA Assays (Applied Biosystems) in a QuantStudio 3 system (Applied Biosystems). For each miR, we used one TaqMan Advanced miRNA Assays with specific probes and primers. miR-16 exhibited the most constant expression levels between the samples and was identified as endogenous control by relative quantification software (Thermo Fisher Scientifc). Based on this and supported with statistical analysis (Supplementary Figure S1) and miR-16 was used as endogenous control. Relative expression levels of the studied miRNAs were calculated using the relative quantification 2^−ΔCT^ method and normalised with miR-16 (ΔCT = CT of target–CT of miR-16). The fold change was calculated by 2^−ΔΔCT^ method using K562 as a reference for cell lines and the expression at diagnosis for patients follow-up.

### Statistical analysis

The statistical analyses were performed using IBM.SPSS®, version 22, except when otherwise indicated. Normal distribution was tested and the following analyses were performed according to these results. Differences between two groups were assessed by nonparametric Mann-Whitney U test and for three or more groups by nonparametric Kruskal-Wallis test followed by Dunn’s multiple comparison tests. The receiver operating characteristic (ROC) curves analysis was performed to evaluate the variables accuracy as biomarkers. For each ROC curve, an optimal cut-off point was determined by the maximum Youden’s J Index, which corresponds to the value of the parameter that maximised the sum of specificity and sensitivity. The positive predictive value (PPV) and negative predictive value (NPV) were calculated for each identified biomarker. The association between profiles and the optimal response was analysed by calculating the odds ratio (OR) and its 95% confidence interval (CI), using Fisher’s exact test with GraphPad Prism version 6.0. G power analysis was performed Gpower software (version 3.1.9.2). The formulas for predicting an optimal response using both miR-21 and miR-451 expression levels were achieved using R software and its general linear model (glm) function with binomial family and logit link, either using continuous or binary miR profile, considering the single effect, main effect, interaction or full factorial models. The Akaike information criterion (AIC’s) minimal value identified the best model. All the statistical tests were considered significant when p < 0.05.

## Supplementary information

Figure S1

## Data Availability

All data generated or analysed during this study are included in this published article.
