# Supplementary material for: MicroRNA signature refine response prediction in CML
Source: Sci Rep. 2019 Jul 4;9:9666. doi: 10.1038/s41598-019-46132-9 (PMC6609611; doi:10.1038/s41598-019-46132-9)
Supplement: Supplementary file 1 — Figure S1 [file 41598_2019_46132_MOESM1_ESM.pdf]

Supplementary Information

MicroRNA signature refine response prediction in CML

Raquel Alves[1,2,3], Ana Cristina Gonçalves[1,2,3], Joana Jorge[1,2,3], Gilberto Marques[4], Dino Luís[5], André B. Ribeiro[2,5], Paulo Freitas-Tavares[5], Bárbara Oliveiros[2,6], António M. Almeida[7,8] and Ana Bela Sarmento-Ribeiro[1,2,3,5*]

Supplementary Figures

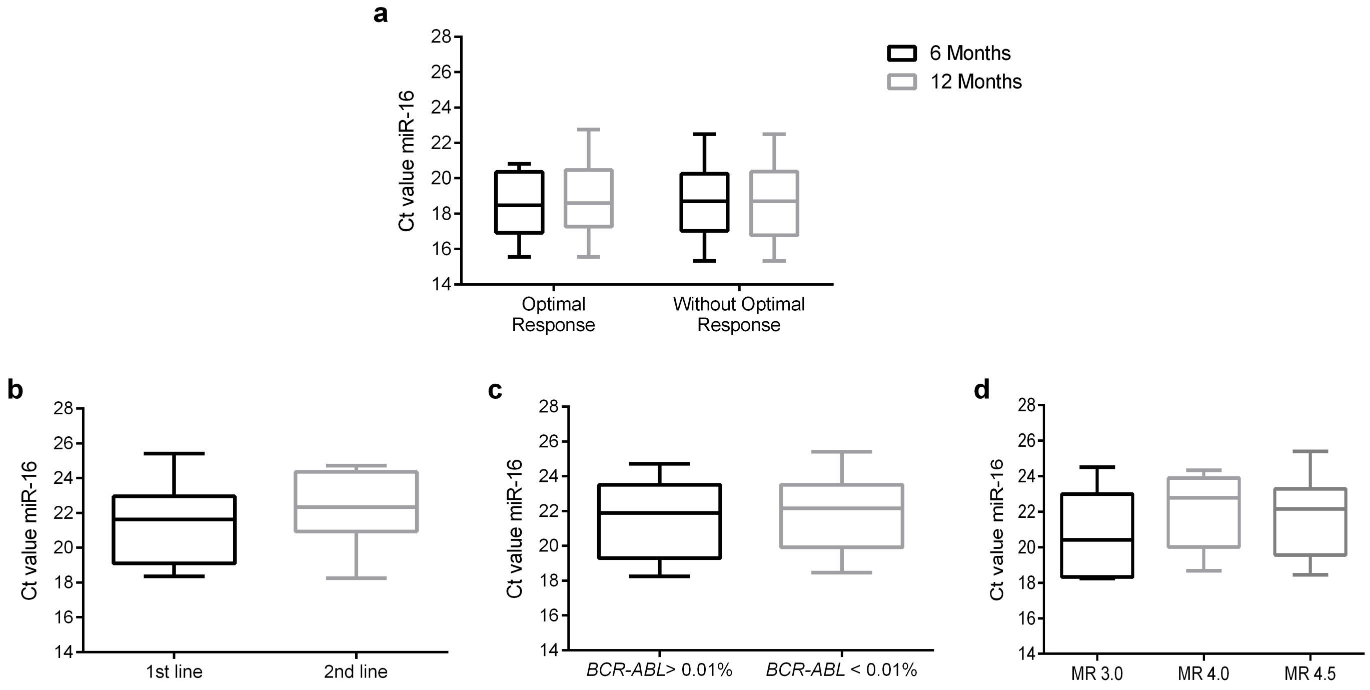

Figure S1 – Ct variance of miR-16 in CML patients according the different comparative groups. The Ct value of miR-16 of CML patients at diagnosis according response levels (a). At follow-up measures, the Ct variance of this miR according to the number of TKI that patients were exposed (b), to *BCR-ABL1* values (c) and to molecular response rates (d). The results are represented as median with interquartile range. Any statistical difference was detected between groups.
